# Bis[μ-2-(2-benzoyl­hydrazinylidenemeth­yl)-6-methoxy­phenolato][2-(2-benzoyl­hydrazinylidenemeth­yl)-6-methoxy­phenolato]dimanganese(II) perchlorate methanol solvate

**DOI:** 10.1107/S1600536810018040

**Published:** 2010-05-22

**Authors:** Gui-Miao Yu, Yun-Hui Li, Li-Fei Zou, Jian-Wei Zhu, Xiao-Qiu Liu

**Affiliations:** a School of Chemistry and Environmental Engineering, Changchun University of Science and Technology, Changchun 130022, People’s Republic of China; b College of Earth Sciences, Jilin University, Changchun 130061, People’s Republic of China

## Abstract

In the title complex, [Mn_2_(C_15_H_13_N_2_O_3_)_3_]ClO_4_·CH_3_OH, the two Mn^II^ ions are bridged by two phenolate O atoms from two ligands, forming an Mn_2_O_2_ quadrangle. Each Mn^II^ ion has a distorted octa­hedral coordination geometry. One Mn^II^ ion is coordinated by two N atoms and four O atoms from two ligands, and the other is coordinated by one N atom and five O atoms from three ligands. A dimer is formed by inter­molecular N—H⋯O hydrogen bonds. The dimers, perchlorate anions and methanol solvent mol­ecules are further connected into a chain along [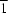
01] through N—H⋯O and O—H⋯O hydrogen bonds.

## Related literature

For general background to the study of Schiff base compounds, see: Ando *et al.* (2004 ▶); Costes *et al.* (1995 ▶); Duda *et al.* (2003 ▶); Siddall *et al.* (1983 ▶). For related structures, see: Li *et al.* (2010 ▶); Huang & Li (2007 ▶); Mikuriya *et al.* (1992 ▶); Yin (2008 ▶); Yu *et al.* (2006 ▶). For the ligand synthesis, see: Pouralimardan *et al.* (2007 ▶); Sacconi (1954 ▶).
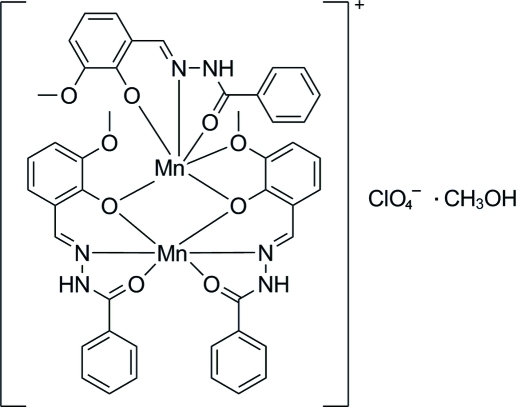


## Experimental

### 

#### Crystal data

[Mn_2_(C_15_H_13_N_2_O_3_)_3_]ClO_4_·CH_4_O
*M* *_r_* = 1049.19
Triclinic, 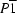

*a* = 12.7184 (6) Å
*b* = 13.8723 (7) Å
*c* = 15.0885 (12) Å
α = 100.268 (1)°
β = 94.030 (1)°
γ = 115.826 (1)°
*V* = 2324.7 (2) Å^3^
*Z* = 2
Mo *K*α radiation
μ = 0.68 mm^−1^
*T* = 173 K
0.15 × 0.12 × 0.10 mm

#### Data collection

Bruker APEXII CCD diffractometer
Absorption correction: multi-scan (*SADABS*; Sheldrick, 1996 ▶) *T*_min_ = 0.906, *T*_max_ = 0.936
11959 measured reflections
8138 independent reflections
6183 reflections with *I* > 2σ(*I*)
*R*_int_ = 0.021

#### Refinement

*R*[*F* ^2^ > 2σ(*F* ^2^)] = 0.045
*wR*(*F* ^2^) = 0.123
*S* = 1.05
8138 reflections
627 parameters
H-atom parameters constrained
Δρ_max_ = 0.49 e Å^−3^
Δρ_min_ = −0.68 e Å^−3^

### 

Data collection: *APEX2* (Bruker, 2007 ▶); cell refinement: *SAINT-Plus* (Bruker, 2007 ▶); data reduction: *SAINT-Plus*; program(s) used to solve structure: *SHELXS97* (Sheldrick, 2008 ▶); program(s) used to refine structure: *SHELXL97* (Sheldrick, 2008 ▶); molecular graphics: *DIAMOND* (Brandenburg, 1999 ▶); software used to prepare material for publication: *SHELXL97* (Sheldrick, 2008 ▶) and *publCIF* (Westrip, 2010 ▶).

## Supplementary Material

Crystal structure: contains datablocks I, global. DOI: 10.1107/S1600536810018040/hy2307sup1.cif

Structure factors: contains datablocks I. DOI: 10.1107/S1600536810018040/hy2307Isup2.hkl

Additional supplementary materials:  crystallographic information; 3D view; checkCIF report

## Figures and Tables

**Table 1:** Selected bond lengths (Å)

Mn1—O2	2.099 (2)
Mn1—O3	2.148 (2)
Mn1—O8	2.105 (2)
Mn1—O9	2.196 (2)
Mn1—N1	2.263 (2)
Mn1—N5	2.253 (3)
Mn2—O1	2.427 (2)
Mn2—O2	2.083 (2)
Mn2—O5	2.061 (2)
Mn2—O6	2.192 (2)
Mn2—O8	2.215 (2)
Mn2—N3	2.200 (3)

**Table 2:** Hydrogen-bond geometry (Å, °)

*D*—H⋯*A*	*D*—H	H⋯*A*	*D*⋯*A*	*D*—H⋯*A*
N2—H2*A*⋯O5^i^	0.88	2.04	2.907 (3)	168
N4—H4*A*⋯O13^ii^	0.88	2.08	2.910 (4)	156
N6—H6*A*⋯O14	0.88	1.98	2.810 (4)	156
O14—H14*A*⋯O10^iii^	0.84	2.05	2.865 (4)	165

Symmetry codes: (i) 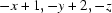; (ii) 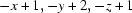; (iii) 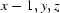.

**Fig. 1.**
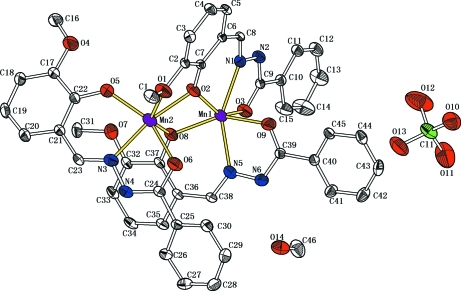
Molecular structure of the title compound. Displacement ellipsoids are drawn at the 30% probability level. H atoms have been omitted for clarity.

**Fig. 2.**
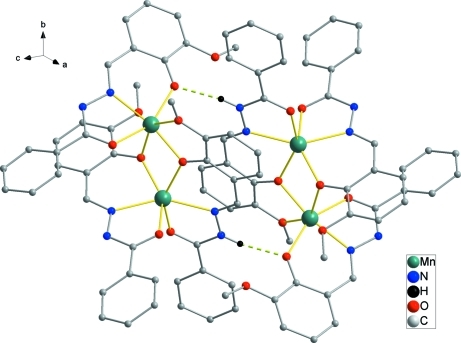
The dimeric structure of the title compound, with hydrogen bonds shown as green dashed lines.

**Fig. 3.**
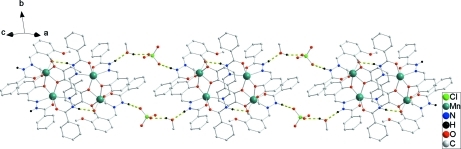
One-dimensional chain structure of the title compound. Hydrogen bonds are shown as dashed lines.

## supplementary crystallographic information

### Comment

Studies of Schiff base compounds are of great interest in various aspects of chemistry, such as homogeneous catalysts in industry, antitumor activities, photoelectric materials, catalytic materials, etc. (Ando *et al.*, 2004; Costes *et al.*, 1995; Duda *et al.*, 2003; Siddall *et al.*, 1983). The crystal structures of metal complexes with salicylaldehyde benzoylhydrazide have been attracted tremendous interest (Huang & Li, 2007; Yin, 2008; Yu *et al.*, 2006). As a continuation of our effort in this system, we investigated a novel Schiff base, 3-methoxysalicylaldehyde benzoylhydrazide (H_2_*L*). This multidentate ligand has several O and N donors with suitable relative positions, which can coordinate to two or more metal centers. In addition, the vanillin group displays a variety of bonding geometries, such as monodentate, chelating, bidentate bridging, monodentate bridging, and chelating bridging (Li *et al.*, 2010). We report here the synthesis and crystal structure of the title compound.

The molecular structure of the title compound is shown in Fig. 1. There are two crystallographically independent Mn^II^ centers with different coordination environments in the asymmetric unit. The two Mn^II^ ions, Mnl and Mn2, are bridged by two phenolate O atoms (O2, O8) from two Schiff base ligands (Table 1). The Mn1···Mn2 separation is 3.284 (1) Å, and the Mn1—O2—Mn2 and Mn1—O8—Mn2 angles are 103.50 (9) and 98.92 (8)°, respectively. The coordination geometry of each Mn^II^ ion is distorted octahedral. The Mn1 atom is coordinated by two N atoms and four O atoms from two ligands. The square plane around the Mn1 atom is formed by O_2_N_2_ donor atoms (N1, N5, O8 and O9) and the axial positions are occupied by phenolate O2 and carbonyl O3. However, the Mn2 atom is coordinated by one N atom and five O atoms from three ligands. The distorted octahedral coordination is achieved by the equatorial plane donor atoms, methoxy O1, carbonyl O2, phenolate O8 and hydrazine N3, and the coordination of phenolate O5 and carbonyl O6 at the axial positions. In addition, the methoxy O1 is weakly bonded to Mn2 with a Mn2—O1 distance of 2.427 (2) Å, which is comparable to those reported for other binuclear Mn^II^ complexes (Mikuriya *et al.*, 1992). In the crystal structure, two adjacent molecules participate in complementary N(hydrazine)—H···O(phenolate) hydrogen bonds, forming a dimeric structure (Fig. 2 and Table 2). The dimers, perchlorate anions and methanol solvent molecules are further connected into a chain structure through N—H···O and O—H···O hydrogen bonds (Fig. 3).

### Experimental

The Schiff base ligand (H_2_*L*) was prepared in a similar manner to the reported procedures (Pouralimardan *et al.*, 2007; Sacconi, 1954). The title compound was synthesized by adding Mn(ClO_4_)_2_.6H_2_O (36.6 mg, 0.1 mmol) and imidazole(6.8 mg, 0.1 mmol) to a solution of H_2_*L* (27.3 mg, 0.1 mmol) in methanol (15 ml). The resulting mixture was stirred for 5 h at room temperature to afford a yellow solution, which was left unperturbed to allow slow evaporation of the solvent. Yellow single crystals suitable for X-ray diffraction analysis were formed after about two weeks.

### Refinement

H atoms were placed in calculated positions and refined using a riding model, with C—H (aromatic) = 0.95 and 0.98 (methyl) Å, N—H = 0.88 Å and O—H = 0.84 Å and with *U*_iso_(H) = 1.2(1.5 for methyl and hydroxy)*U*_eq_(C, N, O).

### Crystal data

**Table:**

[Mn_2_(C_15_H_13_N_2_O_3_)_3_]ClO_4_·CH_4_O	*Z* = 2
*M**_r_* = 1049.19	*F*(000) = 1080
Triclinic, *P*1	*D*_x_ = 1.499 Mg m^−^^3^
Hall symbol: -P 1	Mo *K*α radiation, λ = 0.71073 Å
*a* = 12.7184 (6) Å	Cell parameters from 5673 reflections
*b* = 13.8723 (7) Å	θ = 2.4–25.9°
*c* = 15.0885 (12) Å	µ = 0.68 mm^−^^1^
α = 100.268 (1)°	*T* = 173 K
β = 94.030 (1)°	Block, yellow
γ = 115.826 (1)°	0.15 × 0.12 × 0.10 mm
*V* = 2324.7 (2) Å^3^	

### Data collection

**Table:**

Bruker APEXII CCD diffractometer	8138 independent reflections
Radiation source: fine-focus sealed tube	6183 reflections with *I* > 2σ(*I*)
graphite	*R*_int_ = 0.021
φ and ω scans	θ_max_ = 25.0°, θ_min_ = 1.8°
Absorption correction: multi-scan (*SADABS*; Sheldrick, 1996)	*h* = −15→10
*T*_min_ = 0.906, *T*_max_ = 0.936	*k* = −16→16
11959 measured reflections	*l* = −17→17

### Refinement

**Table:**

Refinement on *F*^2^	Primary atom site location: structure-invariant direct methods
Least-squares matrix: full	Secondary atom site location: difference Fourier map
*R*[*F*^2^ > 2σ(*F*^2^)] = 0.045	Hydrogen site location: inferred from neighbouring sites
*wR*(*F*^2^) = 0.123	H-atom parameters constrained
*S* = 1.05	*w* = 1/[σ^2^(*F*_o_^2^) + (0.0586*P*)^2^ + 1.186*P*] where *P* = (*F*_o_^2^ + 2*F*_c_^2^)/3
8138 reflections	(Δ/σ)_max_ = 0.001
627 parameters	Δρ_max_ = 0.49 e Å^−^^3^
0 restraints	Δρ_min_ = −0.68 e Å^−^^3^

### Fractional atomic coordinates and isotropic or equivalent isotropic displacement parameters (Å^2^)

**Table:**

	*x*	*y*	*z*	*U*_iso_*/*U*_eq_	
Cl1	0.89876 (8)	0.67575 (8)	0.50975 (6)	0.0426 (2)	
Mn1	0.36982 (4)	0.89528 (4)	0.15072 (3)	0.02431 (13)	
Mn2	0.43109 (4)	1.15354 (4)	0.23009 (3)	0.03144 (15)	
N1	0.4807 (2)	0.87719 (19)	0.04411 (16)	0.0228 (5)	
N2	0.4219 (2)	0.77597 (19)	−0.01720 (16)	0.0251 (6)	
H2A	0.4560	0.7548	−0.0600	0.030*	
N3	0.3403 (2)	1.2330 (2)	0.31023 (17)	0.0293 (6)	
N4	0.3060 (2)	1.1885 (2)	0.38472 (18)	0.0322 (6)	
H4A	0.2582	1.2037	0.4174	0.039*	
N5	0.2727 (2)	0.8410 (2)	0.26601 (17)	0.0267 (6)	
N6	0.3164 (2)	0.7859 (2)	0.31286 (17)	0.0294 (6)	
H6A	0.2818	0.7550	0.3559	0.035*	
O1	0.64422 (19)	1.24458 (18)	0.27865 (15)	0.0357 (5)	
O2	0.50670 (18)	1.05637 (16)	0.17192 (15)	0.0293 (5)	
O3	0.26225 (19)	0.74323 (17)	0.05223 (14)	0.0308 (5)	
O4	0.4299 (2)	1.3273 (2)	0.00081 (16)	0.0468 (7)	
O5	0.43845 (19)	1.26231 (17)	0.15090 (14)	0.0312 (5)	
O6	0.42445 (19)	1.10827 (19)	0.36227 (15)	0.0344 (5)	
O7	0.1612 (2)	1.08089 (19)	0.09004 (16)	0.0377 (6)	
O8	0.27945 (18)	0.99182 (17)	0.16429 (14)	0.0287 (5)	
O9	0.46239 (19)	0.82750 (19)	0.22884 (15)	0.0337 (5)	
O10	1.0073 (2)	0.6796 (2)	0.54667 (19)	0.0528 (7)	
O11	0.8019 (3)	0.5765 (2)	0.5162 (3)	0.0818 (11)	
O12	0.9022 (3)	0.6841 (4)	0.4178 (2)	0.0971 (13)	
O13	0.8851 (3)	0.7656 (2)	0.5595 (2)	0.0648 (8)	
O14	0.1488 (2)	0.6610 (2)	0.41134 (19)	0.0510 (7)	
H14A	0.1138	0.6790	0.4513	0.077*	
C1	0.7174 (3)	1.3365 (3)	0.3523 (2)	0.0494 (10)	
H1A	0.7737	1.3195	0.3857	0.074*	
H1B	0.6677	1.3518	0.3938	0.074*	
H1C	0.7609	1.4012	0.3280	0.074*	
C2	0.6994 (3)	1.2000 (3)	0.2201 (2)	0.0273 (7)	
C3	0.8167 (3)	1.2503 (3)	0.2135 (2)	0.0330 (8)	
H3	0.8690	1.3194	0.2531	0.040*	
C4	0.8587 (3)	1.1981 (3)	0.1472 (2)	0.0378 (8)	
H4	0.9399	1.2327	0.1412	0.045*	
C5	0.7840 (3)	1.0981 (3)	0.0912 (2)	0.0310 (7)	
H5	0.8140	1.0643	0.0463	0.037*	
C6	0.6630 (3)	1.0438 (2)	0.0988 (2)	0.0243 (7)	
C7	0.6198 (3)	1.0971 (2)	0.1639 (2)	0.0238 (7)	
C8	0.5897 (3)	0.9368 (2)	0.0389 (2)	0.0244 (7)	
H8	0.6239	0.9091	−0.0066	0.029*	
C9	0.3099 (3)	0.7115 (3)	−0.0082 (2)	0.0270 (7)	
C10	0.2450 (3)	0.6014 (3)	−0.0711 (2)	0.0308 (7)	
C11	0.2905 (3)	0.5623 (3)	−0.1418 (2)	0.0351 (8)	
H11	0.3678	0.6075	−0.1520	0.042*	
C12	0.2250 (4)	0.4588 (3)	−0.1973 (3)	0.0487 (10)	
H12	0.2567	0.4338	−0.2463	0.058*	
C13	0.1160 (4)	0.3924 (4)	−0.1825 (3)	0.0705 (14)	
H13	0.0710	0.3212	−0.2215	0.085*	
C14	0.0703 (4)	0.4277 (4)	−0.1116 (4)	0.0882 (19)	
H14	−0.0052	0.3801	−0.0999	0.106*	
C15	0.1342 (4)	0.5327 (3)	−0.0570 (3)	0.0615 (12)	
H15	0.1011	0.5577	−0.0090	0.074*	
C16	0.4032 (4)	1.3346 (4)	−0.0898 (3)	0.0576 (11)	
H16A	0.3172	1.2961	−0.1099	0.086*	
H16B	0.4408	1.3006	−0.1304	0.086*	
H16C	0.4331	1.4122	−0.0919	0.086*	
C17	0.3771 (3)	1.3615 (3)	0.0659 (2)	0.0399 (9)	
C18	0.3230 (4)	1.4265 (3)	0.0576 (3)	0.0507 (10)	
H18	0.3211	1.4513	0.0030	0.061*	
C19	0.2704 (4)	1.4567 (3)	0.1289 (3)	0.0573 (12)	
H19	0.2341	1.5029	0.1235	0.069*	
C20	0.2720 (4)	1.4192 (3)	0.2055 (3)	0.0509 (10)	
H20	0.2361	1.4398	0.2536	0.061*	
C21	0.3251 (3)	1.3508 (3)	0.2163 (2)	0.0349 (8)	
C22	0.3833 (3)	1.3234 (3)	0.1464 (2)	0.0325 (8)	
C23	0.3085 (3)	1.3055 (3)	0.2952 (2)	0.0357 (8)	
H23	0.2713	1.3307	0.3397	0.043*	
C24	0.3494 (3)	1.1208 (3)	0.4046 (2)	0.0298 (7)	
C25	0.3037 (3)	1.0616 (3)	0.4763 (2)	0.0309 (7)	
C26	0.1919 (3)	1.0360 (3)	0.4984 (2)	0.0372 (8)	
H26	0.1430	1.0614	0.4700	0.045*	
C27	0.1522 (3)	0.9736 (3)	0.5616 (2)	0.0459 (9)	
H27	0.0747	0.9540	0.5752	0.055*	
C28	0.2237 (4)	0.9395 (3)	0.6051 (3)	0.0506 (10)	
H28	0.1958	0.8977	0.6495	0.061*	
C29	0.3351 (4)	0.9651 (3)	0.5852 (3)	0.0496 (10)	
H29	0.3848	0.9421	0.6159	0.060*	
C30	0.3739 (3)	1.0248 (3)	0.5200 (3)	0.0436 (9)	
H30	0.4500	1.0410	0.5047	0.052*	
C31	0.1054 (3)	1.1402 (3)	0.0565 (3)	0.0461 (9)	
H31A	0.0234	1.0889	0.0279	0.069*	
H31B	0.1486	1.1769	0.0112	0.069*	
H31C	0.1059	1.1954	0.1073	0.069*	
C32	0.1105 (3)	1.0228 (3)	0.1532 (2)	0.0317 (8)	
C33	0.0058 (3)	1.0108 (3)	0.1813 (2)	0.0404 (9)	
H33	−0.0358	1.0457	0.1574	0.049*	
C34	−0.0395 (3)	0.9480 (3)	0.2444 (2)	0.0413 (9)	
H34	−0.1120	0.9400	0.2633	0.050*	
C35	0.0195 (3)	0.8980 (3)	0.2794 (2)	0.0352 (8)	
H35	−0.0121	0.8558	0.3229	0.042*	
C36	0.1272 (3)	0.9079 (3)	0.2519 (2)	0.0284 (7)	
C37	0.1759 (3)	0.9730 (3)	0.1888 (2)	0.0273 (7)	
C38	0.1829 (3)	0.8511 (2)	0.2921 (2)	0.0282 (7)	
H38	0.1513	0.8191	0.3408	0.034*	
C39	0.4151 (3)	0.7819 (3)	0.2892 (2)	0.0295 (7)	
C40	0.4667 (3)	0.7237 (3)	0.3367 (2)	0.0304 (7)	
C41	0.4082 (3)	0.6553 (3)	0.3924 (3)	0.0431 (9)	
H41	0.3318	0.6451	0.4031	0.052*	
C42	0.4614 (3)	0.6021 (3)	0.4321 (3)	0.0507 (10)	
H42	0.4210	0.5548	0.4700	0.061*	
C43	0.5727 (4)	0.6170 (3)	0.4175 (3)	0.0477 (10)	
H43	0.6080	0.5789	0.4443	0.057*	
C44	0.6327 (3)	0.6870 (3)	0.3641 (2)	0.0416 (9)	
H44	0.7101	0.6986	0.3550	0.050*	
C45	0.5795 (3)	0.7404 (3)	0.3238 (2)	0.0342 (8)	
H45	0.6207	0.7888	0.2870	0.041*	
C46	0.0717 (4)	0.5557 (4)	0.3563 (4)	0.0767 (15)	
H46A	0.1038	0.5429	0.3007	0.115*	
H46B	−0.0064	0.5515	0.3398	0.115*	
H46C	0.0640	0.4996	0.3902	0.115*	

### Atomic displacement parameters (Å^2^)

**Table:**

	*U*^11^	*U*^22^	*U*^33^	*U*^12^	*U*^13^	*U*^23^
Cl1	0.0341 (5)	0.0521 (6)	0.0478 (5)	0.0259 (4)	0.0126 (4)	0.0075 (4)
Mn1	0.0245 (3)	0.0270 (3)	0.0250 (3)	0.0148 (2)	0.0078 (2)	0.00504 (19)
Mn2	0.0328 (3)	0.0336 (3)	0.0319 (3)	0.0191 (2)	0.0108 (2)	0.0045 (2)
N1	0.0270 (14)	0.0203 (13)	0.0225 (13)	0.0124 (11)	0.0055 (11)	0.0036 (10)
N2	0.0286 (15)	0.0252 (14)	0.0216 (13)	0.0137 (12)	0.0065 (11)	0.0013 (11)
N3	0.0305 (15)	0.0319 (15)	0.0262 (14)	0.0150 (12)	0.0094 (12)	0.0046 (12)
N4	0.0356 (16)	0.0368 (16)	0.0322 (15)	0.0218 (13)	0.0167 (13)	0.0086 (12)
N5	0.0258 (14)	0.0299 (14)	0.0271 (14)	0.0159 (12)	0.0062 (11)	0.0043 (11)
N6	0.0303 (15)	0.0369 (16)	0.0291 (14)	0.0194 (13)	0.0112 (12)	0.0136 (12)
O1	0.0300 (13)	0.0326 (13)	0.0369 (13)	0.0115 (10)	0.0088 (10)	−0.0036 (10)
O2	0.0227 (12)	0.0262 (12)	0.0380 (13)	0.0121 (10)	0.0108 (10)	0.0002 (10)
O3	0.0291 (12)	0.0310 (12)	0.0310 (12)	0.0140 (10)	0.0096 (10)	0.0015 (10)
O4	0.0661 (18)	0.0687 (18)	0.0340 (13)	0.0499 (16)	0.0220 (13)	0.0228 (13)
O5	0.0357 (13)	0.0332 (12)	0.0311 (12)	0.0197 (11)	0.0111 (10)	0.0095 (10)
O6	0.0314 (13)	0.0426 (14)	0.0345 (13)	0.0213 (11)	0.0101 (10)	0.0082 (11)
O7	0.0361 (13)	0.0443 (14)	0.0446 (14)	0.0265 (12)	0.0085 (11)	0.0157 (12)
O8	0.0234 (11)	0.0343 (12)	0.0337 (12)	0.0171 (10)	0.0087 (10)	0.0084 (10)
O9	0.0299 (13)	0.0480 (14)	0.0355 (13)	0.0237 (11)	0.0135 (10)	0.0205 (11)
O10	0.0397 (15)	0.0699 (19)	0.0589 (17)	0.0322 (14)	0.0091 (13)	0.0198 (15)
O11	0.0371 (17)	0.0475 (18)	0.150 (3)	0.0131 (14)	0.0151 (19)	0.014 (2)
O12	0.098 (3)	0.190 (4)	0.0464 (19)	0.100 (3)	0.0219 (18)	0.033 (2)
O13	0.0533 (18)	0.0531 (17)	0.095 (2)	0.0311 (15)	0.0346 (17)	0.0086 (16)
O14	0.0425 (15)	0.0571 (18)	0.0637 (18)	0.0250 (14)	0.0296 (14)	0.0238 (14)
C1	0.048 (2)	0.051 (2)	0.036 (2)	0.019 (2)	0.0038 (18)	−0.0115 (18)
C2	0.0303 (18)	0.0305 (17)	0.0271 (16)	0.0190 (15)	0.0068 (14)	0.0070 (14)
C3	0.0256 (18)	0.0294 (18)	0.0377 (19)	0.0100 (15)	−0.0008 (15)	0.0016 (15)
C4	0.0227 (18)	0.042 (2)	0.049 (2)	0.0153 (16)	0.0102 (16)	0.0070 (17)
C5	0.0264 (18)	0.0385 (19)	0.0347 (18)	0.0210 (16)	0.0093 (14)	0.0063 (15)
C6	0.0265 (17)	0.0291 (17)	0.0238 (16)	0.0173 (14)	0.0069 (13)	0.0082 (13)
C7	0.0214 (16)	0.0285 (17)	0.0248 (16)	0.0138 (14)	0.0050 (13)	0.0073 (13)
C8	0.0275 (17)	0.0295 (17)	0.0234 (16)	0.0189 (14)	0.0084 (13)	0.0065 (13)
C9	0.0298 (18)	0.0278 (17)	0.0268 (17)	0.0163 (15)	0.0053 (14)	0.0064 (13)
C10	0.0296 (18)	0.0285 (17)	0.0333 (18)	0.0132 (15)	0.0053 (14)	0.0047 (14)
C11	0.036 (2)	0.0324 (19)	0.0339 (19)	0.0155 (16)	0.0062 (15)	0.0014 (15)
C12	0.051 (2)	0.040 (2)	0.049 (2)	0.021 (2)	0.0105 (19)	−0.0056 (18)
C13	0.057 (3)	0.041 (2)	0.079 (3)	0.004 (2)	0.014 (2)	−0.019 (2)
C14	0.055 (3)	0.052 (3)	0.104 (4)	−0.010 (2)	0.031 (3)	−0.024 (3)
C15	0.046 (2)	0.043 (2)	0.070 (3)	0.005 (2)	0.023 (2)	−0.012 (2)
C16	0.076 (3)	0.079 (3)	0.040 (2)	0.050 (3)	0.019 (2)	0.021 (2)
C17	0.046 (2)	0.046 (2)	0.040 (2)	0.0293 (19)	0.0168 (17)	0.0145 (17)
C18	0.072 (3)	0.058 (3)	0.047 (2)	0.046 (2)	0.021 (2)	0.024 (2)
C19	0.086 (3)	0.060 (3)	0.059 (3)	0.059 (3)	0.023 (2)	0.022 (2)
C20	0.073 (3)	0.053 (2)	0.048 (2)	0.045 (2)	0.022 (2)	0.0129 (19)
C21	0.040 (2)	0.0361 (19)	0.0350 (19)	0.0234 (17)	0.0111 (16)	0.0069 (15)
C22	0.0320 (19)	0.0300 (18)	0.0377 (19)	0.0164 (15)	0.0082 (15)	0.0065 (15)
C23	0.041 (2)	0.0362 (19)	0.0359 (19)	0.0247 (17)	0.0124 (16)	0.0029 (15)
C24	0.0245 (17)	0.0315 (18)	0.0279 (17)	0.0111 (14)	0.0031 (14)	−0.0010 (14)
C25	0.0309 (18)	0.0310 (18)	0.0292 (17)	0.0134 (15)	0.0093 (14)	0.0035 (14)
C26	0.034 (2)	0.042 (2)	0.0322 (18)	0.0165 (17)	0.0067 (15)	0.0046 (16)
C27	0.036 (2)	0.052 (2)	0.041 (2)	0.0116 (18)	0.0108 (17)	0.0122 (18)
C28	0.061 (3)	0.046 (2)	0.046 (2)	0.021 (2)	0.017 (2)	0.0185 (19)
C29	0.057 (3)	0.053 (2)	0.052 (2)	0.033 (2)	0.011 (2)	0.022 (2)
C30	0.044 (2)	0.050 (2)	0.046 (2)	0.0287 (19)	0.0134 (18)	0.0125 (18)
C31	0.042 (2)	0.043 (2)	0.062 (3)	0.0269 (19)	0.0021 (19)	0.0165 (19)
C32	0.0263 (18)	0.0353 (19)	0.0351 (18)	0.0183 (15)	0.0033 (15)	0.0013 (15)
C33	0.034 (2)	0.056 (2)	0.042 (2)	0.0321 (19)	0.0059 (16)	0.0061 (18)
C34	0.0263 (19)	0.055 (2)	0.045 (2)	0.0235 (18)	0.0099 (16)	0.0029 (18)
C35	0.0274 (18)	0.0365 (19)	0.0384 (19)	0.0139 (16)	0.0096 (15)	0.0014 (15)
C36	0.0223 (16)	0.0312 (17)	0.0291 (17)	0.0137 (14)	0.0036 (13)	−0.0026 (14)
C37	0.0229 (17)	0.0287 (17)	0.0276 (17)	0.0138 (14)	0.0021 (13)	−0.0045 (13)
C38	0.0241 (17)	0.0288 (17)	0.0287 (17)	0.0109 (14)	0.0077 (14)	0.0015 (14)
C39	0.0311 (18)	0.0306 (17)	0.0274 (17)	0.0149 (15)	0.0054 (14)	0.0063 (14)
C40	0.0318 (18)	0.0296 (17)	0.0315 (18)	0.0159 (15)	0.0055 (14)	0.0065 (14)
C41	0.036 (2)	0.049 (2)	0.049 (2)	0.0191 (18)	0.0078 (17)	0.0239 (19)
C42	0.042 (2)	0.053 (2)	0.060 (3)	0.019 (2)	0.0020 (19)	0.030 (2)
C43	0.059 (3)	0.043 (2)	0.050 (2)	0.032 (2)	−0.003 (2)	0.0135 (19)
C44	0.045 (2)	0.051 (2)	0.038 (2)	0.0332 (19)	−0.0008 (17)	0.0027 (17)
C45	0.040 (2)	0.041 (2)	0.0289 (18)	0.0257 (17)	0.0073 (15)	0.0061 (15)
C46	0.054 (3)	0.058 (3)	0.120 (4)	0.028 (3)	0.023 (3)	0.016 (3)

### Geometric parameters (Å, °)

**Table:**

Cl1—O12	1.414 (3)	C12—C13	1.351 (6)
Cl1—O11	1.418 (3)	C12—H12	0.9500
Cl1—O13	1.422 (3)	C13—C14	1.368 (6)
Cl1—O10	1.428 (3)	C13—H13	0.9500
Mn1—O2	2.099 (2)	C14—C15	1.381 (6)
Mn1—O3	2.148 (2)	C14—H14	0.9500
Mn1—O8	2.105 (2)	C15—H15	0.9500
Mn1—O9	2.196 (2)	C16—H16A	0.9800
Mn1—N1	2.263 (2)	C16—H16B	0.9800
Mn1—N5	2.253 (3)	C16—H16C	0.9800
Mn2—O1	2.427 (2)	C17—C18	1.367 (5)
Mn2—O2	2.083 (2)	C17—C22	1.419 (5)
Mn2—O5	2.061 (2)	C18—C19	1.403 (5)
Mn2—O6	2.192 (2)	C18—H18	0.9500
Mn2—O8	2.215 (2)	C19—C20	1.351 (5)
Mn2—N3	2.200 (3)	C19—H19	0.9500
Mn1—Mn2	3.284 (1)	C20—C21	1.407 (5)
N1—C8	1.285 (4)	C20—H20	0.9500
N1—N2	1.383 (3)	C21—C22	1.420 (4)
N2—C9	1.344 (4)	C21—C23	1.430 (5)
N2—H2A	0.8800	C23—H23	0.9500
N3—C23	1.284 (4)	C24—C25	1.473 (4)
N3—N4	1.384 (4)	C25—C26	1.388 (5)
N4—C24	1.343 (4)	C25—C30	1.388 (5)
N4—H4A	0.8800	C26—C27	1.376 (5)
N5—C38	1.289 (4)	C26—H26	0.9500
N5—N6	1.377 (3)	C27—C28	1.372 (5)
N6—C39	1.350 (4)	C27—H27	0.9500
N6—H6A	0.8800	C28—C29	1.372 (6)
O1—C2	1.388 (4)	C28—H28	0.9500
O1—C1	1.429 (4)	C29—C30	1.378 (5)
O2—C7	1.320 (3)	C29—H29	0.9500
O3—C9	1.249 (4)	C30—H30	0.9500
O4—C17	1.358 (4)	C31—H31A	0.9800
O4—C16	1.418 (4)	C31—H31B	0.9800
O5—C22	1.322 (4)	C31—H31C	0.9800
O6—C24	1.242 (4)	C32—C33	1.375 (4)
O7—C32	1.372 (4)	C32—C37	1.424 (4)
O7—C31	1.430 (4)	C33—C34	1.391 (5)
O8—C37	1.320 (4)	C33—H33	0.9500
O9—C39	1.242 (4)	C34—C35	1.361 (5)
O14—C46	1.410 (5)	C34—H34	0.9500
O14—H14A	0.8400	C35—C36	1.416 (4)
C1—H1A	0.9800	C35—H35	0.9500
C1—H1B	0.9800	C36—C37	1.417 (5)
C1—H1C	0.9800	C36—C38	1.445 (4)
C2—C3	1.364 (4)	C38—H38	0.9500
C2—C7	1.404 (4)	C39—C40	1.483 (4)
C3—C4	1.401 (5)	C40—C45	1.383 (5)
C3—H3	0.9500	C40—C41	1.385 (5)
C4—C5	1.365 (5)	C41—C42	1.377 (5)
C4—H4	0.9500	C41—H41	0.9500
C5—C6	1.412 (4)	C42—C43	1.380 (5)
C5—H5	0.9500	C42—H42	0.9500
C6—C7	1.410 (4)	C43—C44	1.376 (5)
C6—C8	1.444 (4)	C43—H43	0.9500
C8—H8	0.9500	C44—C45	1.384 (5)
C9—C10	1.480 (4)	C44—H44	0.9500
C10—C15	1.373 (5)	C45—H45	0.9500
C10—C11	1.385 (4)	C46—H46A	0.9800
C11—C12	1.374 (5)	C46—H46B	0.9800
C11—H11	0.9500	C46—H46C	0.9800
			
O12—Cl1—O11	111.2 (3)	C12—C13—H13	120.0
O12—Cl1—O13	108.7 (2)	C14—C13—H13	120.0
O11—Cl1—O13	108.39 (19)	C13—C14—C15	119.8 (4)
O12—Cl1—O10	108.54 (18)	C13—C14—H14	120.1
O11—Cl1—O10	109.91 (18)	C15—C14—H14	120.1
O13—Cl1—O10	110.12 (18)	C10—C15—C14	120.9 (4)
O2—Mn1—O8	76.87 (8)	C10—C15—H15	119.6
O2—Mn1—O3	145.50 (8)	C14—C15—H15	119.6
O8—Mn1—O3	109.15 (8)	O4—C16—H16A	109.5
O2—Mn1—O9	98.66 (9)	O4—C16—H16B	109.5
O8—Mn1—O9	143.09 (8)	H16A—C16—H16B	109.5
O3—Mn1—O9	95.20 (9)	O4—C16—H16C	109.5
O2—Mn1—N5	119.63 (9)	H16A—C16—H16C	109.5
O8—Mn1—N5	79.31 (9)	H16B—C16—H16C	109.5
O3—Mn1—N5	94.72 (9)	O4—C17—C18	125.1 (3)
O9—Mn1—N5	71.21 (8)	O4—C17—C22	113.2 (3)
O2—Mn1—N1	77.63 (8)	C18—C17—C22	121.7 (3)
O8—Mn1—N1	128.89 (9)	C17—C18—C19	120.5 (4)
O3—Mn1—N1	72.54 (8)	C17—C18—H18	119.7
O9—Mn1—N1	84.16 (8)	C19—C18—H18	119.7
N5—Mn1—N1	151.29 (9)	C20—C19—C18	119.1 (3)
O5—Mn2—O2	109.55 (9)	C20—C19—H19	120.4
O5—Mn2—O6	150.92 (8)	C18—C19—H19	120.4
O2—Mn2—O6	96.68 (8)	C19—C20—C21	122.2 (3)
O5—Mn2—N3	82.02 (9)	C19—C20—H20	118.9
O2—Mn2—N3	168.30 (9)	C21—C20—H20	118.9
O6—Mn2—N3	72.45 (9)	C20—C21—C22	119.4 (3)
O5—Mn2—O8	107.68 (8)	C20—C21—C23	117.0 (3)
O2—Mn2—O8	74.83 (8)	C22—C21—C23	123.5 (3)
O6—Mn2—O8	90.80 (8)	O5—C22—C17	118.9 (3)
N3—Mn2—O8	100.50 (9)	O5—C22—C21	124.1 (3)
O5—Mn2—O1	92.92 (9)	C17—C22—C21	117.0 (3)
O2—Mn2—O1	68.95 (8)	N3—C23—C21	124.6 (3)
O6—Mn2—O1	84.77 (8)	N3—C23—H23	117.7
N3—Mn2—O1	113.22 (9)	C21—C23—H23	117.7
O8—Mn2—O1	142.67 (7)	O6—C24—N4	120.1 (3)
C8—N1—N2	117.0 (2)	O6—C24—C25	121.8 (3)
C8—N1—Mn1	131.2 (2)	N4—C24—C25	118.1 (3)
N2—N1—Mn1	111.15 (17)	C26—C25—C30	118.8 (3)
C9—N2—N1	116.7 (2)	C26—C25—C24	123.2 (3)
C9—N2—H2A	121.6	C30—C25—C24	117.9 (3)
N1—N2—H2A	121.6	C27—C26—C25	119.8 (3)
C23—N3—N4	117.4 (3)	C27—C26—H26	120.1
C23—N3—Mn2	130.8 (2)	C25—C26—H26	120.1
N4—N3—Mn2	111.61 (18)	C28—C27—C26	120.5 (4)
C24—N4—N3	116.6 (2)	C28—C27—H27	119.7
C24—N4—H4A	121.7	C26—C27—H27	119.7
N3—N4—H4A	121.7	C29—C28—C27	120.7 (4)
C38—N5—N6	116.8 (3)	C29—C28—H28	119.7
C38—N5—Mn1	130.1 (2)	C27—C28—H28	119.7
N6—N5—Mn1	113.08 (18)	C28—C29—C30	119.0 (4)
C39—N6—N5	116.4 (3)	C28—C29—H29	120.5
C39—N6—H6A	121.8	C30—C29—H29	120.5
N5—N6—H6A	121.8	C29—C30—C25	121.2 (4)
C2—O1—C1	118.0 (3)	C29—C30—H30	119.4
C2—O1—Mn2	111.43 (18)	C25—C30—H30	119.4
C1—O1—Mn2	130.5 (2)	O7—C31—H31A	109.5
C7—O2—Mn2	122.82 (18)	O7—C31—H31B	109.5
C7—O2—Mn1	133.25 (18)	H31A—C31—H31B	109.5
Mn2—O2—Mn1	103.50 (9)	O7—C31—H31C	109.5
C9—O3—Mn1	118.2 (2)	H31A—C31—H31C	109.5
C17—O4—C16	118.2 (3)	H31B—C31—H31C	109.5
C22—O5—Mn2	133.1 (2)	O7—C32—C33	125.0 (3)
C24—O6—Mn2	114.7 (2)	O7—C32—C37	113.9 (3)
C32—O7—C31	117.1 (3)	C33—C32—C37	121.1 (3)
C37—O8—Mn1	131.7 (2)	C32—C33—C34	120.5 (3)
C37—O8—Mn2	116.67 (18)	C32—C33—H33	119.8
Mn1—O8—Mn2	98.92 (8)	C34—C33—H33	119.8
C39—O9—Mn1	118.4 (2)	C35—C34—C33	120.3 (3)
C46—O14—H14A	109.5	C35—C34—H34	119.8
O1—C1—H1A	109.5	C33—C34—H34	119.8
O1—C1—H1B	109.5	C34—C35—C36	120.9 (3)
H1A—C1—H1B	109.5	C34—C35—H35	119.5
O1—C1—H1C	109.5	C36—C35—H35	119.5
H1A—C1—H1C	109.5	C35—C36—C37	119.6 (3)
H1B—C1—H1C	109.5	C35—C36—C38	117.0 (3)
C3—C2—O1	125.1 (3)	C37—C36—C38	123.4 (3)
C3—C2—C7	122.3 (3)	O8—C37—C36	123.1 (3)
O1—C2—C7	112.5 (3)	O8—C37—C32	119.3 (3)
C2—C3—C4	118.8 (3)	C36—C37—C32	117.5 (3)
C2—C3—H3	120.6	N5—C38—C36	124.2 (3)
C4—C3—H3	120.6	N5—C38—H38	117.9
C5—C4—C3	120.6 (3)	C36—C38—H38	117.9
C5—C4—H4	119.7	O9—C39—N6	120.1 (3)
C3—C4—H4	119.7	O9—C39—C40	120.6 (3)
C4—C5—C6	121.3 (3)	N6—C39—C40	119.3 (3)
C4—C5—H5	119.4	C45—C40—C41	119.6 (3)
C6—C5—H5	119.4	C45—C40—C39	117.0 (3)
C7—C6—C5	118.3 (3)	C41—C40—C39	123.4 (3)
C7—C6—C8	123.4 (3)	C42—C41—C40	119.7 (4)
C5—C6—C8	118.3 (3)	C42—C41—H41	120.1
O2—C7—C2	118.6 (3)	C40—C41—H41	120.1
O2—C7—C6	122.7 (3)	C41—C42—C43	120.6 (4)
C2—C7—C6	118.7 (3)	C41—C42—H42	119.7
N1—C8—C6	123.4 (3)	C43—C42—H42	119.7
N1—C8—H8	118.3	C44—C43—C42	120.1 (3)
C6—C8—H8	118.3	C44—C43—H43	120.0
O3—C9—N2	120.9 (3)	C42—C43—H43	120.0
O3—C9—C10	120.8 (3)	C43—C44—C45	119.5 (3)
N2—C9—C10	118.3 (3)	C43—C44—H44	120.3
C15—C10—C11	118.0 (3)	C45—C44—H44	120.3
C15—C10—C9	117.9 (3)	C40—C45—C44	120.5 (3)
C11—C10—C9	124.0 (3)	C40—C45—H45	119.7
C12—C11—C10	120.7 (3)	C44—C45—H45	119.7
C12—C11—H11	119.6	O14—C46—H46A	109.5
C10—C11—H11	119.6	O14—C46—H46B	109.5
C13—C12—C11	120.4 (4)	H46A—C46—H46B	109.5
C13—C12—H12	119.8	O14—C46—H46C	109.5
C11—C12—H12	119.8	H46A—C46—H46C	109.5
C12—C13—C14	120.1 (4)	H46B—C46—H46C	109.5

### Hydrogen-bond geometry (Å, °)

**Table:**

*D*—H···*A*	*D*—H	H···*A*	*D*···*A*	*D*—H···*A*
N2—H2A···O5^i^	0.88	2.04	2.907 (3)	168
N4—H4A···O13^ii^	0.88	2.08	2.910 (4)	156
N6—H6A···O14	0.88	1.98	2.810 (4)	156
O14—H14A···O10^iii^	0.84	2.05	2.865 (4)	165

Symmetry codes: (i) −*x*+1, −*y*+2, −*z*; (ii) −*x*+1, −*y*+2, −*z*+1; (iii) *x*−1, *y*, *z*.
